# Ruptured Cornual Ectopic Pregnancy Mimics as Acute Retention of Urine: A Case Report

**DOI:** 10.7759/cureus.86398

**Published:** 2025-06-19

**Authors:** Kandiah Guruparan, R Hemabala

**Affiliations:** 1 Department of Obstetrics and Gynaecology, Faculty of Medicine, University of Jaffna, Jaffna, LKA; 2 Department of Health, Teaching Hospital Jaffna, Jaffna, LKA

**Keywords:** ectopic pregnancy, en masse lesion, ruptured cornual ectopic, unusual presentation, urinary retention

## Abstract

Cornual ectopic pregnancy is a rare entity of extrauterine pregnancies and once ruptured, it is associated with high maternal morbidity and mortality. Most of the extrauterine pregnancies are detected with early pregnancy ultrasound scan, but there are diagnostic difficulties in the early detection of cornual ectopic pregnancy. Early recognition of unruptured cornual ectopic and interventions are associated with better outcomes. We report a case of successfully managed ruptured cornual ectopic pregnancy, which presented as acute retention of urine to the emergency unit at the Teaching Hospital, Jaffna.

## Introduction

Cornual pregnancy accounts for 2%-4% of ectopic pregnancies in contrast to ampullary ectopic pregnancies (80%) [1]. It is located within the uterine musculature outside the uterine cavity. The cornual part of the fallopian tube is in the uterine horn wall musculature and measures 1.2 cm in length and 0.7 cm in width. Though ultrasound is the mainstay of diagnosis in extrauterine pregnancy, there are diagnostic difficulties in the early detection of cornual ectopic pregnancy [2-4]. Any delay in diagnosis is associated with rupture of cornual ectopic with several complications such as severe hemorrhage and shock [5,6].

## Case presentation

A 33-year-old para 2 was transferred from a peripheral hospital with acute retention of urine and abdominal distension for one-day duration. She had irregular menstrual cycles and could not recall her last menstrual period. On admission, she was clinically pale and tachycardic. Her pulse rate and blood pressure were 110/min and 90/60 mmHg, respectively. Her abdominopelvic examination revealed a right-sided pelvic mass to the size of 14-week gravid uterus with cervical motion tenderness. Her urine pregnancy test was positive and serum beta-human chorionic gonadotropin (β-hCG) was 3029 miu/ml (Table 1). Her haemoglobin was 9 g/dl (reference range: 12-16 g/dl).

**Table 1:** Serum beta hCG (miu/ml) reference for weeks of gestation Serum beta-hCG levels were measured using the Elecsys HCG+β (Roche Diagnostics, Rotkreuz, Switzerland) assay via Cobas e-analyzer (Roche Diagnostics) (ECLIA method) [7]. hCG: human chorionic gonadotropin; ECLIA: electrochemiluminescence immunoassay.

Weeks of gestation	Median (miu/ml)	5th centile (miu/ml)	95th centile (miu/ml)
3	17.5	5.8	72.2
4	141	9.5	750
5	1,398	217	7138
6	3,339	158	31,795
7	39,759	3,697	163,563
8	90,084	32,065	149,571
9	106,257	63,803	151,410
10	85,172	46,509	186,977
12	66,676	27,832	210,612
14	34,440	13,950	62,630

A departmental radiology scan revealed an empty uterine cavity with a large well-defined mixed echogenic mass (7.2x9.8x7.3 cm) in the pouch of Douglas. A full bladder was also noted on scan. A ruptured ectopic pregnancy with organized haematoma was considered as the first differential diagnosis. (Unfortunately, we did not have the ultrasound image.)

**Figure 1:**
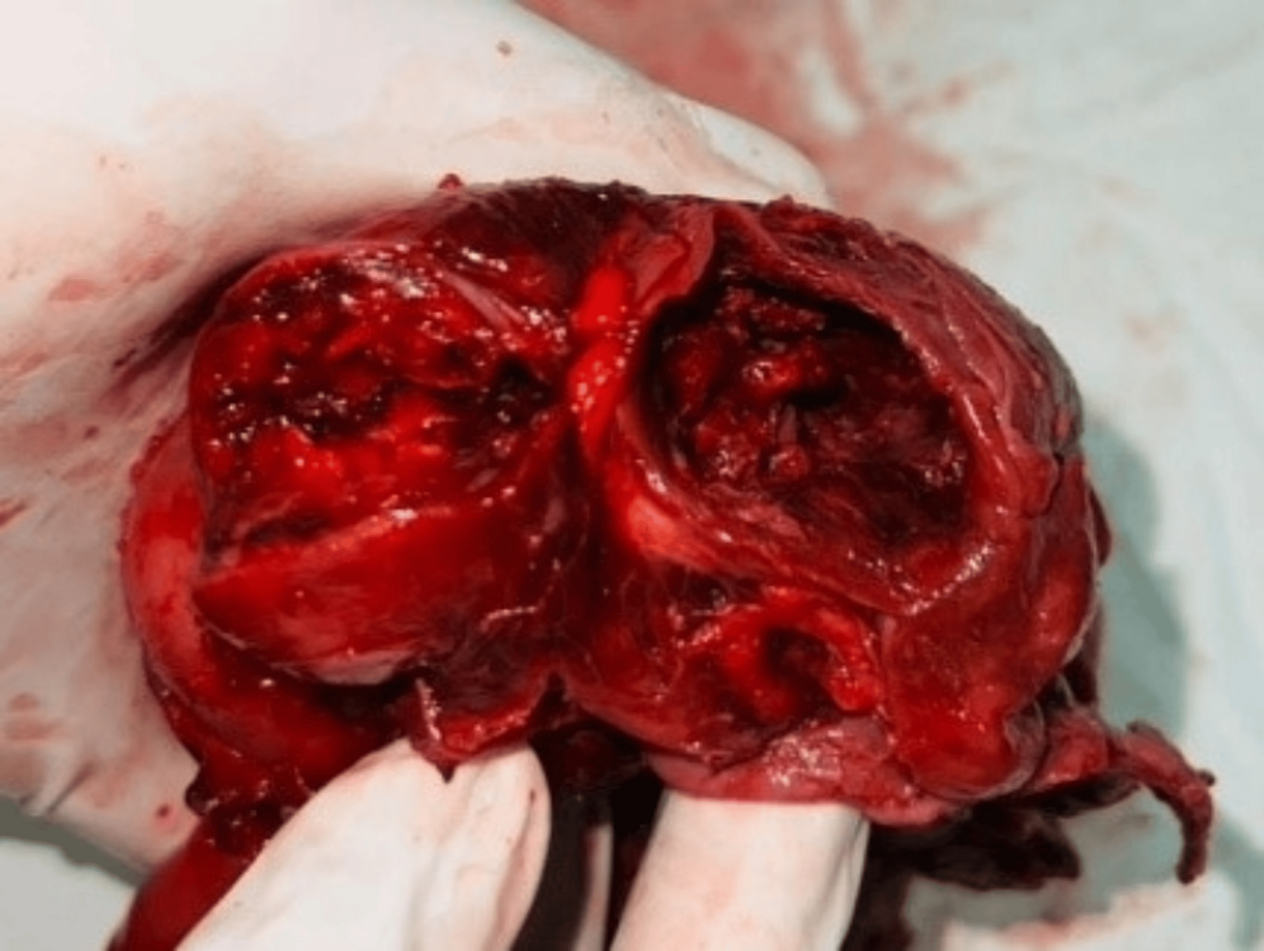
En masse lesion at surgery. Uterus, ovaries, and tubes were seen as en masse at laparotomy.

In view of her clinical condition, she was catheterized and proceeded with laparotomy. During surgery, the bladder was found to be adhered to the anterior abdominal wall and a well-defined necrotic mass was seen in the pelvis (10x10x10 cm). Uterus, tubes, and ovaries cannot be separated and so it was an ‘en masse’ lesion in the pelvis totally covered by the omentum (Figure 1). There was no ascites or hemoperitoneum in the pelvis. The fragile mass was carefully dissected out and proceeded with hysterectomy.

A fortnight prior to the current admission, she was treated for acute urinary retention conservatively. A routine urine pregnancy test was weakly positive during that admission. Therefore, evaluation with serial serum beta hCG and scan were planned as there was no period of gestation or no ultrasound features of intrauterine pregnancy. She defaulted from the follow-up until the current hospital admission.

**Figure 2:**
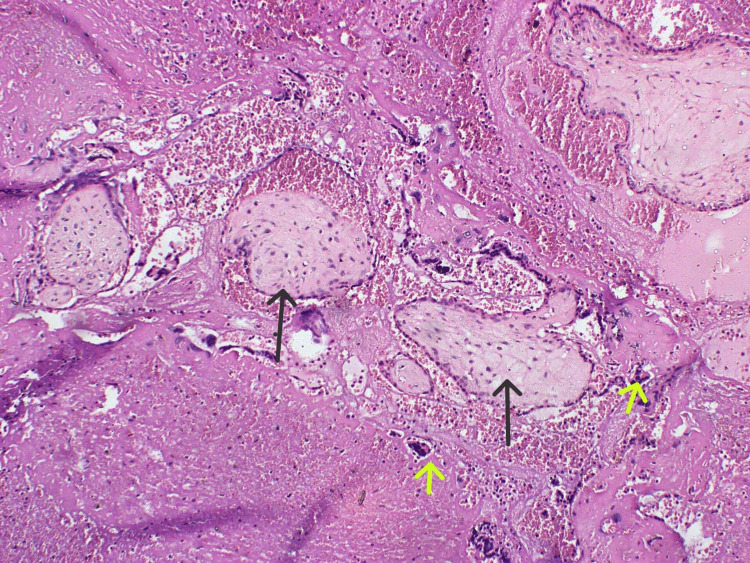
Histopathology of the cornual ectopic. Black arrow indicates chorionic villi. Yellow arrow indicates trophoblastic cells.

Histopathological findings confirmed a distorted mass consisting of uterus with cystic mass in the posterior aspect of uterus, cervix, and left tubo-ovarian mass macroscopically and a right cornual ectopic pregnancy with adhered hemorrhagic cyst with right adnexal soft tissue (Figure 2).

## Discussion

Even though ultrasound scan plays an important role in detecting ectopic pregnancy early, cornual ectopic could be missed or diagnosed later when compared to other extrauterine pregnancies. The majority of cornual ectopic present as acute emergencies with features of shock following rupture [8,9].

The management of cornual ectopic depends on the case presentation, clinical condition, periods of gestation, and the available resources. Unruptured cornual ectopic with serum beta hCG less than 1500 iu could be managed with systemic or local methotrexate in clinically stable patients with no contraindication for methotrexate. Mostly, cornual ectopic can be managed surgically either via laparoscopy or laparotomy [3,4].

In our case, the patient presented initially with acute retention of urine and was managed conservatively. As her urine pregnancy test was weakly positive, the patient was advised to follow up with an ultrasound scan and serial beta hCG assessments. But she did not turn to the clinic for follow-up.

Two weeks later from the initial admission, she was admitted to the emergency unit with severe abdominal pain and urinary retention. As her clinical, biochemical, and imaging assessments led to the diagnosis of ruptured ectopic pregnancy, she underwent emergency laparotomy and a well-sealed mass was found in the pelvis.

Though the patient had a ruptured extrauterine pregnancy, she was fortunate to survive due to the well-sealed en masse effect and clotting mechanism. This could be the reason for her unusual clinical presentation of this patient. She did not have significant internal bleeding or its adverse consequences. The entire necrotic mass had to be removed as it was difficult to differentiate the tubes, ovaries, and part of the uterus during the surgery. The patient had an uneventful recovery.

## Conclusions

Despite advancements in the early diagnosis of cornual ectopic pregnancy, clinicians face challenges in managing it. Ruptured cornual ectopic pregnancies are associated with high morbidities and mortalities as well as massive blood transfusions. Clinical suspicion and close monitoring with ultrasound and serum beta hCG assessments improve the outcomes. In under-resource settings, encouraging patients to attend the clinic follow-ups is important and involving the field health team (medical officers of health and field midwives) is vital.
